# The Challenge of Planning Conservation Strategies in Threatened Seascapes: Understanding the Role of Fine Scale Assessments of Community Response to Cumulative Human Pressures

**DOI:** 10.1371/journal.pone.0149253

**Published:** 2016-02-12

**Authors:** Giuseppe Guarnieri, Stanislao Bevilacqua, Francesco De Leo, Giulio Farella, Anna Maffia, Antonio Terlizzi, Simonetta Fraschetti

**Affiliations:** Department of Biological and Environmental Sciences and Technologies, University of Salento, CoNISMa, 73100, Lecce, Italy; Seoul National University, REPUBLIC OF KOREA

## Abstract

Assessing the distribution and intensity of human threats to biodiversity is a prerequisite for effective spatial planning, harmonizing conservation purposes with sustainable development. In the Mediterranean Sea, the management of Marine Protected Areas (MPAs) is rarely based on explicit consideration of the distribution of multiple stressors, with direct assessment of their effects on ecosystems. This gap limits the effectiveness of protection and is conducive to conflicts among stakeholders. Here, a fine scale assessment of the potential effects of different combinations of stressors (both land- and marine-based) on vulnerable rocky habitats (i.e. lower midlittoral and shallow infralittoral) along 40 km of coast in the western Mediterranean (Ionian Sea) has been carried out. The study area is a paradigmatic example of socio-ecological interactions, where several human uses and conservation measures collide. Significant differences in the structure of assemblages according to different combinations of threats were observed, indicating distinct responses of marine habitats to different sets of human pressures. A more complex three-dimensional structure, higher taxon richness and β-diversity characterized assemblages subject to low versus high levels of human pressure, consistently across habitats. In addition, the main drivers of change were: closeness to the harbour, water quality, and the relative extension of beaches. Our findings suggest that, although efforts to assess cumulative impacts at large scale may help in individuating priority areas for conservation purposes, the fact that such evaluations are often based on expert opinions and not on actual studies limits their ability to represent real environmental conditions at local scale. Systematic evaluations of local scale effects of anthropogenic drivers of change on biological communities should complement broad scale management strategies to achieve effective sustainability of human exploitation of marine resources.

## Introduction

A combination of escalating pressures related to human activities is threatening almost all marine ecosystems worldwide [1], leading to increasing interest towards the assessment of the effects of multiple stressors to develop management tools conciliating conservation targets with social and economic development [2]. Understanding how coastal-marine space is used, and how human pressures interact with natural drivers of change ultimately affecting marine ecosystems is a priority for effective management [3–7].

In the Mediterranean Sea, the distribution of cumulative threats to marine ecosystems has been dealt with at basin scale [8,9]. These analyses move beyond the traditional single-threat approach, identifying key threats to different components of biodiversity and allowing site prioritization for different uses, while considering complex scenarios. This approach has been also applied at more restricted scales, by examining the expected outcomes of alternative management scenarios [7,10]. Despite these advances, the interpretation of environmental changes caused by the compound effects of multiple anthropogenic disturbances is often based on by expert judgment only [11,12], with poor validation with empirical data on ecosystem components [13]. The potential to fully understand the effects of multiple, co-occurring stressors should rely on more ecologically realistic approaches [14,15] accounting for the heterogeneity of impacts across space and time, the non-linear response of ecosystems to anthropogenic pressures, and a deeper understanding about thresholds and tipping points.

The detailed mapping of human pressures at relevant spatial scales for resource conservation and management, combined with direct quantitative assessments of the effects of multiple stressors in the field, may be conducive to understand how different combinations of stressors interact within communities [6,16,17]. Attempts in this direction are still rare [13], and this is the first application of this approach in the Mediterranean basin. The study was carried out in a densely populated area including the Marine Protected Area (MPA) of Porto Cesareo (SW, Apulia, Italy) established in 1997, comprising sites of community importance (SCIs) aimed to regulate multiple human pressures on *Posidonia oceanica* seagrass meadows, a critical habitat for the EU Habitats Directive. Despite this, the whole area has been, and still is, subjected to several human uses producing impacts whose effects have been investigated by single-threat approaches [18–20]. In this context we combined a fine scale assessment on the distribution and intensity of the human threats occurring in the area with a quantitative evaluation of their effects on two Mediterranean rocky habitats (i.e. lower midlittoral, shallow infralittoral) along 40 km of coast.

The outputs of the study identify the challenges and the gaps of knowledge to be addressed to understand the complexity of relationships between human activities and their ecological impacts on coastal environment, in order to guide future management actions.

## Materials and Methods

### Study area

**Fig 1:**
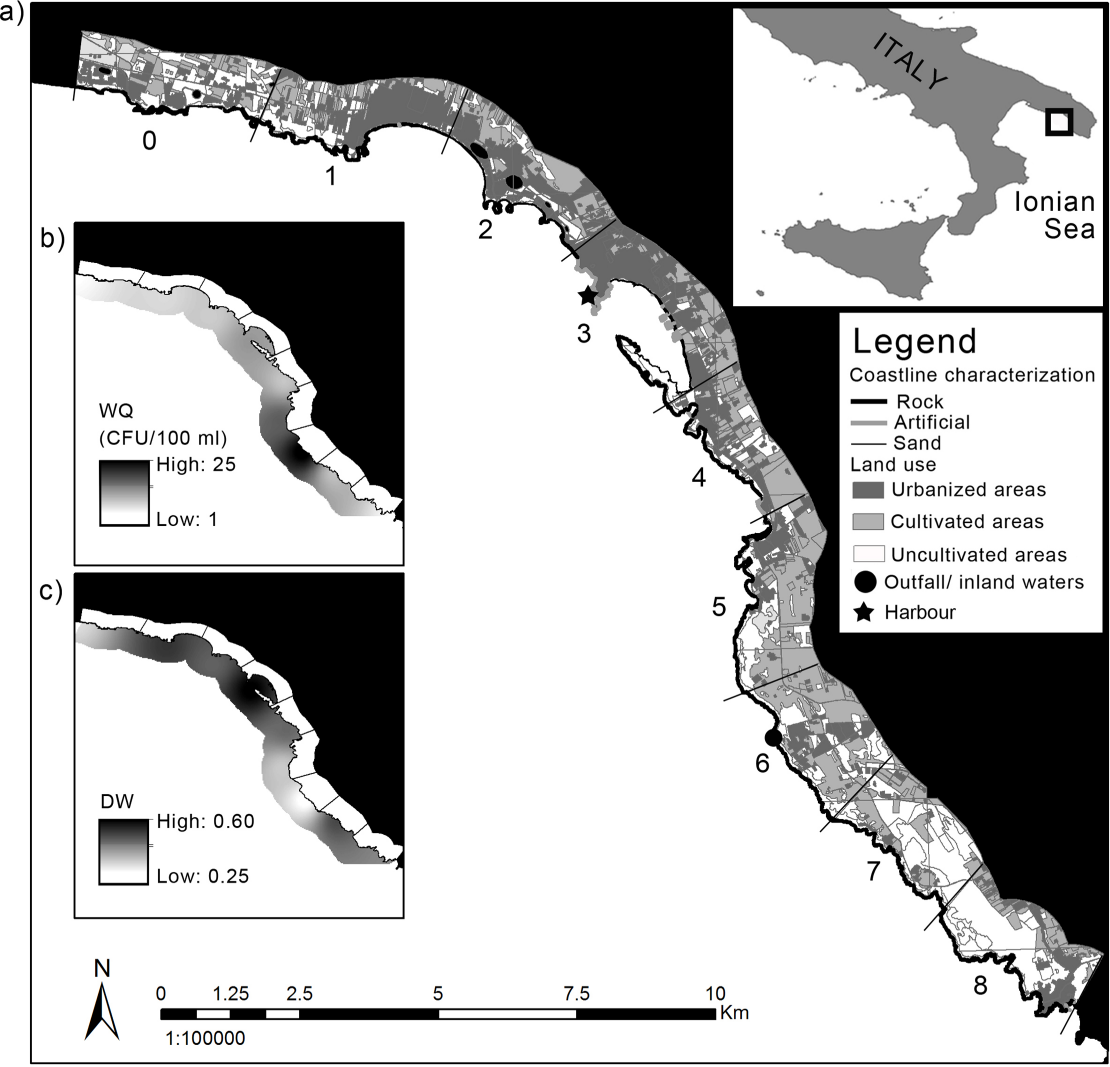
Pressure regimes along the surveyed shore. Map of the study area and pressure regimes: a) land-based threat indicators; b) and c) sea-based threat indicators. WQ = water quality; DW = weighted index of rock damage due to date-mussel collection (see text for details). Numbers from 0 to 8 indicate sectors. Maps were created using ArcGIS® 10.1 software by ESRI (Environmental Systems Resource Institute, www.esri.com). Land use data derived from the Territorial Information System of Apulia Region (www.sitpuglia.it).

The study has been carried out along 40 km of coastline in the Ionian Sea, SW Apulia (Fig 1) and partially overlaps with the MPA of Porto Cesareo (40°14’35”N—17°54’07”E), one of the largest Italian marine reserves (16654 ha). Three SCIs, covering a surface of 7169 ha, are included in the MPA. A gently sloping calcareous rocky plateau almost entirely characterizes the whole area to about 10 m depth; the waters are oligotrophic, due to low concentrations of chlorophyll a (i.e. 0.17–0.50 μg L^-1^) [21]. The shallow infralittoral reefs characterizing the area have been historically depleted by the date mussel collection [18,19], and the effects of this destructive practice (banned since 1988) are still evident along the whole surveyed coast, notwithstanding the establishment of the MPA in 1997. According to official data coming from the Italian Statistic Institute (ISTAT), in 2012 the resident population in the area was 5507 inhabitants (159 inhabitants km^-2^) reaching 73578 presences (approximately 2100 persons km^-2^) due to tourism (source: http://www.agenziapugliapromozione.it) in summer time (from June to September).

### Sampling activities and experimental design

The entire shoreline was digitalized and the relative amounts of sandy, rocky, and artificial coast were assessed on the basis of orthophotos using GIS software (ArcGIS 10.1). This allowed the identification of nine adjacent sectors (from 0 to 8, Fig 1A) of approximately 4 km of coastal straight length each. Sectors had different coastal lengths to ensure that, in each of them, rocky bottoms featured at least the 50% of the coastline. In each sector, three sampling sites (100s m apart from each other) were randomly selected for the surveys of benthic assemblages on rocky reefs from the lower midlittoral and the shallow infralittoral. All sites were characterized by similar substrate complexity, slope, and wave exposure. At each site, the percentage cover of individual sessile taxa was visually estimated within four randomly selected areas (i.e. replicates) covering a surface of ∼ 0.5 m^2^ each. This was done directly in the field for the lower midlittoral (depth comprised between—0.1 to 0.1 m across mean-low-level water) by mean of 12 adjacent quadrats (20 × 20 cm). Due to the conspicuous presence of the brown algae *Cystoseira* spp., the percentage cover of understory assemblage was visually estimated after the cover evaluation of this canopy-forming species and its removal. In the shallow infralittoral (∼7 m depth, slope of the bottom ∼30%), assemblages were sampled through photographic methods. At each site, 12 digital photographic samples (16 × 25 cm) were taken and analysed later by using images editing software.

In both cases, the cover of sessile organisms was estimated and values expressed as percentage of total sampled area. Organisms that were not easily identifiable at species level were lumped into higher taxonomic groups or into morphological groups (see S1 Table for details). Sampling was carried out in spring-summer 2013. The Porto Cesareo MPA Authority issued all permits needed for performing fieldwork.

### Assessment of the distribution of threats

The formal quantification of threats for each coastal sector was carried out through the collection of georeferenced data on a set of indicators of drivers potentially affecting the studied coastal ecosystems (S2 Table). Environmental data were elaborated and visualized using ArcGIS and Marine Geospatial Ecology Tools [22]. High-resolution land use data were obtained from the Territorial Information System of Apulia Region (www.sitpuglia.it). Specifically, because of their recognized role in affecting coastal environments [23], two land-based indicators were taken into account, namely the proportion of urbanized (URB) and cultivated areas (AGR). URB has been chosen as a proxy of coastal modification (i.e. urban areas), demographic pressure (e.g. population density), and non-point sources of urban pollution (e.g. run-off waters from buildings, streets and sidewalks). AGR was assumed as a proxy of land-based pollution related to pesticide inputs and nutrient enrichment through terrestrial run-off. To obtain standardized values, the extent of urbanized and cultivated areas was determined within a buffer area of 1 km inland from the coast for each sector, and expressed as the ratio to the total area of the buffer.

The same information system was used to describe substrate features of the coast (i.e. sandy, rocky and artificial). In particular, the proportion of sandy coasts (SC), besides providing information on geomorphological features of the coast, was considered an indicator of high erosion rates (http://www.eurosion.org) as a consequence of coastal modification (e.g. presence of man-made structures along the shoreline, beach replenishments). Indeed, in the last decade, beach replenishment has been carried out regularly to guarantee tourist frequentation during summer. Therefore, the proportion of sandy bottom could be considered a proxy of indirect effects of human interference with natural sedimentation regimes, likely affecting neighbouring rocky assemblages by increasing sandy bottom instability, sediment load, and water turbidity [24,25].

The distance from the harbour (HD) was employed as a proxy of pressures deriving from maritime traffic, such as boating, anchoring, non-professional or artisanal fishing practices. HD was calculated as the linear distance (km) between the harbour and the centre of each sector. GIS analyses highlighted that modified coastline characterized almost exclusively the harbour zone (sector 3, Fig 1A). Therefore, HD may also represent a proxy of potential influence of artificial structures (e.g. artificial reefs, pontoons, marinas) [26] on natural rocky reef assemblages.

The analyses considered two sea-based indicators: water quality (WQ) and the damage of date mussel collection (DW). Information on water quality was derived from available data on faecal contamination of coastal waters (considered variable: CFU of *Escherichia coli* in 100 ml of sea water) during the period of highest tourist frequentation (i.e. from April to September) collected in 2013 by the Regional Agency for Environmental Prevention and Protection (ARPA; www.arpa.puglia.it). For the analysis, the highest value recorded during the entire monitoring activity (always recorded between July and August) was adopted for each sector. WQ was considered as a proxy of coastal point sources of sewage discharge (i.e. outfalls or waste water inputs coming from inland artificial basins, Fig 1A).

For each sector, DW, the impact of the date mussel *Lithophaga lithophaga* collection was assessed in five randomly selected areas of 10 × 2 m at 5–7 m depth. This allowed to obtain a weighted index of rock damage (DW) ranging between 0 (absence of damage) and 1 (complete desertification) based on the size and frequency of disturbed patches in each sampled surface (for details see [18]). As for WQ, the highest value recorded in each sector was considered. This variable strongly affects connectivity and resilience potential of the shallow rocky shore assemblages [19,27].

Additional variables that could have been used as indicators of human pressures, such as chlorophyll, nitrogen, and phosphorous concentration, or intensity of professional fisheries, were not taken into account because the spatial resolution of available data was not compatible with the spatial scales considered in this study, or because data were not georeferenced. However, several indicators considered in the analysis were likely correlated with the trophic status of the investigated systems (e.g., URB, AGR, WQ). As far as professional fishing activities, trawling is not allowed within the MPA whilst artisanal fisheries (mainly trammel net and long lines) are widespread in the whole area. A recent project mapping artisanal fisheries showed that they occur at least at 5 km far from the sites selected in this study, thus excluding direct effects on the investigated habitats. By contrast, indirect outcomes, such as those related to the removal of top fish predators and cascading effects were likely to occur consistently in all sectors [28].

### Statistical analyses

Data on human pressures were normalized and a Principal Component Analysis (PCA) coupled with a CLUSTER analysis based on Euclidean distance among sectors were carried out in order to identified groups of sectors exposed to comparable levels and combinations of the different threats. When among-sector dissimilarity was ≤ 50% sectors were clustered together. Both CLUSTER and PCA analyses allowed the identification of five groups of sectors, each group corresponding to a different combination of threats.

Two separate distance-based permutational multivariate analyses of variance (PERMANOVA) [29], one for each of the two investigated habitats, were performed to test for differences in the structure of rocky benthic assemblages among the sector groups identified by the PCA and CLUSTER analyses. PERMANOVAs were based on Bray-Curtis dissimilarities calculated on untransformed data, and each term was tested using 4999 random permutations. For both PERMANOVAs, the experimental design consisted of 3 factors: Threat (Th, 5 levels, fixed), Sector (Se, from 1 to 3 levels, random and nested in Th), Site (Si, 3 levels, random and nested in Se), with n = 4. The number of levels of sectors within threat combination actually differed; however, formal tests were still possible since PERMANOVA allows the handling of complex unbalanced designs. For both habitats, a Canonical Analysis of Principal Coordinates (CAP) [30] based on the Bray-Curtis dissimilarity matrix among sites was performed for the term Th in order to visualize patterns of difference in assemblage structure among threat combinations. Individual taxa that might be responsible for any group differences seen in the CAP plots were investigated by calculating product–moment correlations of original variables (taxa) with canonical axes [30]. These correlations of individual variables with the two canonical axes (*r*_1_ and *r*_2_) were then represented as lines in the CAP plots. Taxa were included in the plot only if exceeding an arbitrarily chosen value of correlation (i.e. $\sqrt{r_{1}^{2}+r_{2}^{2}}$ ≥ 0.5).

Permutational Analysis of Multivariate Dispersion (PERMDISP) on the basis of Jaccard dissimilarity was used to test potential variation in β-diversity among threat combinations at the scale of replicates [31] for both habitats. This approach allowed comparisons of small-scale patchiness (in terms of species composition) in assemblages characterized by different combinations of pressures. Moreover, for both data sets, ANOVA was employed to test for differences in species richness (i.e. number of taxa) among threat combinations. ANOVA was also performed to test for differences in the percentage cover of algae *Cystoseira* spp. to investigate the effects of human stressors might have on this canopy forming species [32]. Post hoc pair-wise comparisons were used to test, for each variable, differences among the mean values detected in each threat combination. The design for ANOVAs was the same adopted for PERMANOVA.

The BEST procedure was employed to identify environmental variables that best matched with the biological data, in terms of multivariate structure of assemblages [33].

All analyses were performed using the computer program PRIMER version 6, including the add-on package PERMANOVA+ [34].

## Results

### Threat analysis

As reported in Fig 1A, the highest urbanization characterized sectors 1, 2 and 3 in the northern part of the coastline, where urban areas represent almost the 40% of the coast (up to the 58% in sector 3). The extension of cultivated areas was higher in sectors 4, 5, 6. Sector 6 was featured by the lowest water quality (i.e. the highest value of WQ) (Fig 1B and 1C). Values estimating the rock damage on shallow infralittoral due to data-mussel collection (i.e. DW) highlighted widespread barrens along the entire subtidal seascape of the investigated area. However, the highest frequency of disturbed patches was observed in sectors 2, 3, 7, and 8 (Fig 1C). The northern part of the considered coastline (from sector 0 to 3) was featured by large extents of beaches compared to southern sectors, featured by more continuous rocky substrates.

PCA and CLUSTER analyses identified five groups of sectors, characterized by a consistent occurrence and intensity of threats (T), namely T1 (Sector 2), T2 (Sectors 0, 1, 3), T3 (Sectors 4, 5), T4 (Sector 6), and T5 (Sectors 7, 8) (S1A Fig). The proximity to the harbour (HD), high level of urbanization (URB) and the largest extension of sandy shores (SC) combined with moderate extension of cultivated areas (AGR) characterize T1. Moderate water quality (WQ) and high rock damage due to date mussel collection (DW) also characterize this stretch of coast. T2 was featured by the occurrence of a similar combination of threats, although with generally lower levels with the exception of URB and DW, featured by the highest value for both threats. A decrease in SC, DW and URB combined with a general increase of AGR and WQ (i.e. increasing extension of cultivated areas and decreasing of water quality respectively) contributed to differentiate the remaining threat combinations (i.e. from T3 to T5), with a general lowest level for each threat found in T5 and the worse water quality condition in T4.

### Structure of benthic assemblages

**Fig 2:**
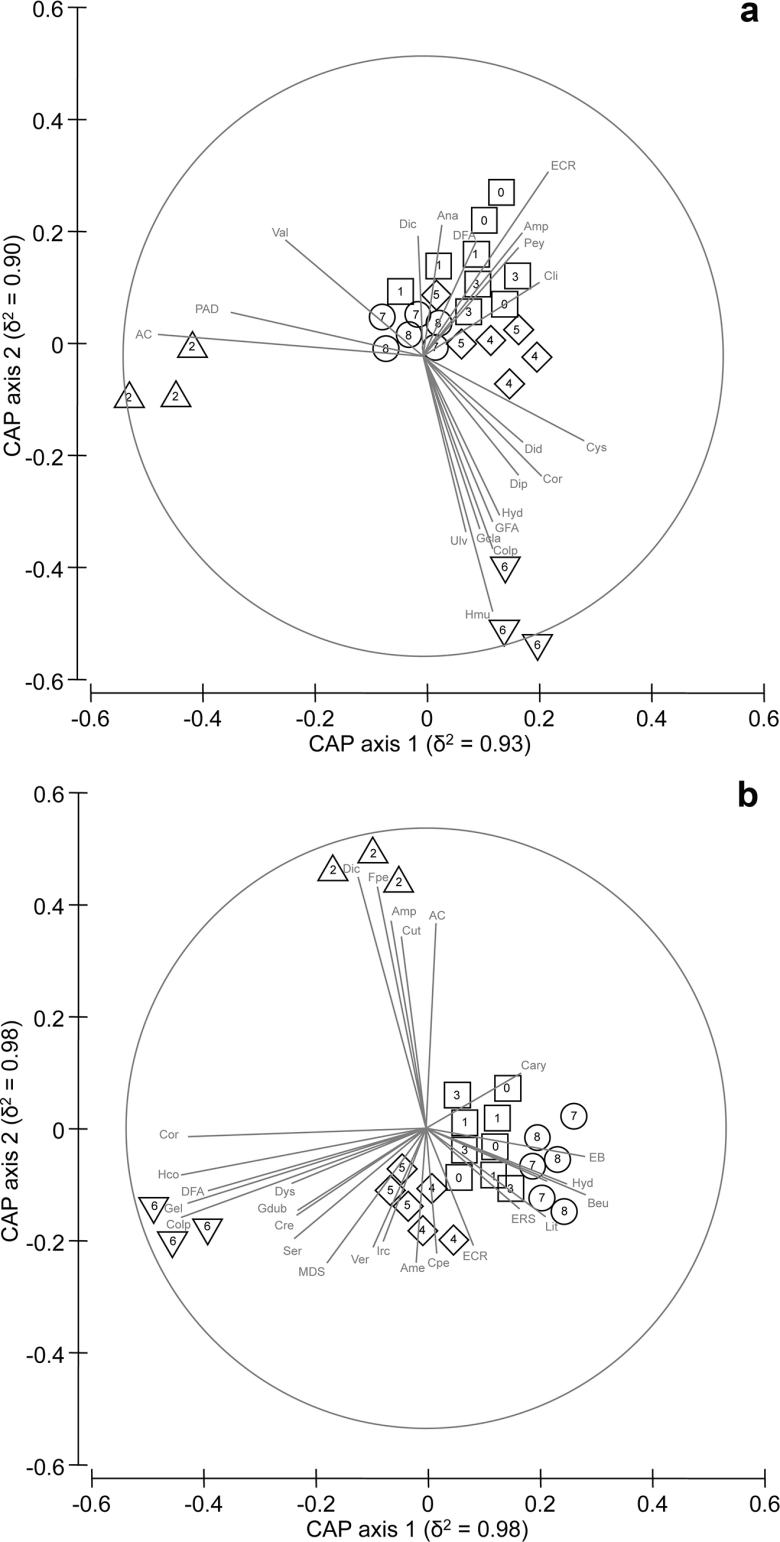
Canonical analysis of principal coordinates on rocky bottom assemblages for different threat combinations. CAP for the factor Th based on the distance matrix of sites of (a) lower midlittoral and (b) shallow infralittoral. △ = threat combination 1 (T1); □ = threat combination 2 (T2); ◇ = threat combination 3 (T3); ▽ = threat combination 4 (T4); ○ = threat combination 5 (T5). Numbers indicates sectors belonging to each threat combination (see S1A Fig for details). Individual taxa highly correlated with canonical axes are shown. Abbreviations for taxa used in CAP plots are given in S1 Table.

PERMANOVA showed significant differences in the structure of assemblages according to different combinations of threat. For both habitats, a significant variability at the scale of sectors was also observed (Table 1A). CAP plots showed the clustering of sectors characterized by the same occurrence and levels of threats, and a clear separation of those sectors grouped in T1 and T4 from the others. This pattern was consistent in both habitats (Fig 2A and 2B). More specifically, turf-forming species (such as articulated corallines, *Padina pavonica*, *Amphiroa rigida* and Dictyotales), and algae typical of eutrophic waters (such as *Ulva* sp., *Hypnea musciformis*, *Colpomenia sinuosa* and algae belonging to the order Gelidiales) mostly contributed to differentiate respectively T1 and T4 from the other groups. Among invertebrates, suspension/filter feeders, such as polichaetes (mainly Serpulids), and a suite of sponges (e.g. *Hemimycale columella*, massive dark sponges [MDS], *Chondrosia reniformis* and *Dysidea avara*) also contributed in structuring the shallow infralittoral assemblages of T4 with respect to the others along the coast (Fig 2B). As far as the other sectors in T2, T3, T5 instead, taxa mostly contributing to characterize sessile assemblages varied between the two habitats. In the lower midlittoral (Fig 2A), sectors within T2 were mostly characterized by a combination of turf-forming (e.g. *Amphiroa rigida*, *Anadyomene stellata*, dark filamentous algae [DFA]) and encrusting algae (encrusting calcifed rhodophytes [ECR], *Peyssonnelia* spp.*)*, whereas sectors within T3 and T5 were featured by more structured assemblages, with taxa typical of the understory of *Cystoseira* canopy characterizing T3 (e.g. Didemnidae, *Diplosoma listerianum*, Clionidae). In the shallow infralittoral (Fig 2B) taxa typical of barren grounds (e.g. ECR, *Balanophyllia europaea*, *Caryophyllia* sp., encrusting bryozoans [EB] and encrusting red sponges [ERS]) characterized assemblages of sectors of T2, T3 and T5, although the presence of massive sponges (e.g. *Ircinia variabilis*, massive dark sponges [MDS]) further contributed to characterize T3 with respect to the other groups with different combinations of threats.

**Table 1:** a) Summary of PERMANOVA testing for the effect of different human pressures acting in the different sectors along the coast on assemblages characterizing the lower midlittoral and the shallow infralittoral. b) Summary of BEST analysis to assess the contribution of a set of environmental variables (stressors) to changes in rocky assemblages.

a)		**Lower midlittoral**			**Shallow infralittoral**		
Source of variation	df	MS	Pseudo-*F*	*P*(perm)	MS	Pseudo-*F*	*P*(perm)
Th = Threat	4	22570	2.84	0.0212	20089	1.89	0.034
Se(Th) = Sector	4	7952	6.14	0.0002	10612	8.95	0.0002
Si(Se(Th)) = Site	18	1296	1.46	0.011	1186	0.99	0.5158
Residuals	81	889			1197		
Total	107						
b)	**Lower midlittoral**		**Shallow infralittoral**				
Environmental variables	Corr.	Sign.	Corr.	Sign.			
URB	0.21	*	0.09	NS			
AGR	0.15	*	0.18	*			
HD	0.43	**	0.29	**			
SC	0.53	**	0.51	**			
WQ	0.31	*	0.46	**			
DW	0.06	NS	0.20	*			
**Best matching**	**SC;HD;WQ(0.66)**		**SC;HD;WQ(0.68)**				

PERMANOVA analyses were based on Bray-Curtis dissimilarities, and each test was performed using 4999 permutation of appropriate units. BEST analyses were based on Spearman's correlation ρ (with 999 permutations). Best matching environmental variables and the overall correlation values (in brackets) are given in bold.

* *p < 0*.*05*

** p < 0.01

NS = Not Significant. Corr. = Correlation (ρ), Sign. = Significance. For environmental variable acronyms see S2 Table.

**Fig 3:**
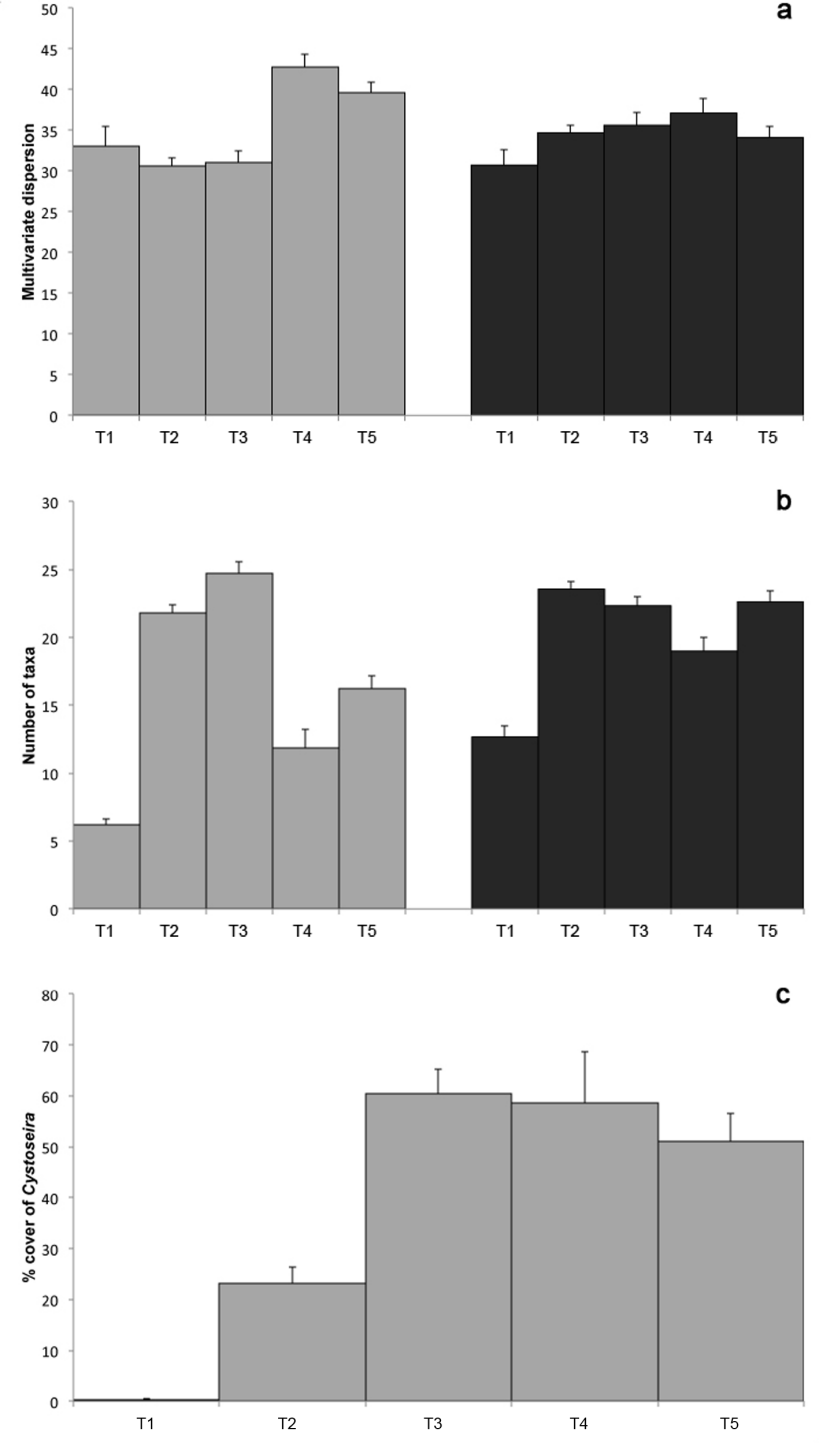
Variation in spatial heterogeneity, species richness and *Cystoseira* coverage across different threat combinations. a) Average (± SE) multivariate dispersion based on Jaccard dissimilarity matrices of both habitat assemblages (i.e. small-scale patchiness) across threat combinations (from T1 to T5); b) mean n° of Taxa (± SE) observed in each sector for both habitats; c) Mean percentage cover (± SE) of *Cystoseira* spp. canopy recorded in the lower midlittoral within each sector. White bars = Lower midlittoral; Black bars = Shallow infralittoral.

The PERMDISP analysis showed significant differences for the term Threat in the lower midlittoral (*F* = 13.1, *p* = 0.001), indicating that the patchiness of species distribution changed significantly among sectors featured by a different occurrence and intensity of threats. More specifically, as reported in Fig 3A, for this habitat the lowest values were detected for T1, T2 and T3. By contrast, no differences in small-scale patchiness were detected in the shallow infralittoral (Fig 3A).

ANOVA on the total number of taxa showed significant differences among sectors characterized by the same occurrence and intensity of threats (respectively: *F* = 7.96, *p* < 0.05 in the lower midlittoral; *F* = 49.13, *p* < 0.01 in the shallow infralittoral). Post-hoc pairwise comparisons showed the lowest values for T1 and T4, consistently across the two habitats (Fig 3B). By contrast, in the remaining sectors, independently from the presence of different combination of threats, the average number of taxa was comparable.

The ANOVA carried out to assess differences in the abundance of *Cystoseira* spp. on lower midlittoral did not detect differences among groups of sectors featured by different threat combinations. However, Fig 3C suggests that these algae were dominant from T3 to T5 (i.e. corresponding to the southernmost part of the coast), where the percentage cover is always higher than 50% compared to the others. Such canopy forming species were absent from T1.

### Correlation between threats and community patterns

BEST analysis (Table 1B) carried out on the threat proxies showed the best matching for the SC, WQ, HD combined, with SC showing the higher correlation value with the pattern of variation of assemblages in both habitats. Two groups of sectors were identified by CLUSTER analysis (S1B Fig), according to the proportion of sandy surface characterizing each sector. Sectors localized in the north (from 0 to 3 [i.e. T1 and T2]) were featured by a higher proportion of sandy coast (from 20 to 50%) with respect to the others (from 5 to 8 [i.e. T3, T4 and T5]), which accounted for larger extensions of rocky reefs.

## Discussion

This is one of the first attempts to quantify the combined effect of co-occurring threats across different habitats. Despite variations in patterns of spatial distribution and intensity among the examined threats along the investigated stretch of coast, clusters of coastal sectors characterized by specific combinations and levels of human pressures were evident. Threat combinations T1 and T2 encompassed medium-to-high levels of pressure for almost all the considered threats, whereas T3-4-5 showed medium-to-low levels, although T4 exhibited the worse condition for water quality. Significant differences in the structure of assemblages associated to distinct combinations of threats were detected, indicating a strong correspondence between the overall level of human pressure and the observed response of marine communities. No attempts to weight threats based on their potential impact on the investigated habitats has been made, since data on the magnitude of the effects of different combination of threats are still largely lacking for most Mediterranean habitats. Therefore, we assumed the effects of different threats as comparable. Although this assumption may be unrealistic in some cases, studies aimed at weighting the potential impact of different anthropogenic drivers provided very close scores for the threats considered here, at least for lower midlittoral and the shallow infralittoral rocky habitats [35], suggesting that weighting would have little influence on our findings.

Distance from the harbor (HD), water quality (WQ) and the extension of sandy shores (SC) all concurred in best explaining differences in assemblages associated to distinct threat combinations, consistently in both habitats, suggesting that harbour activities, sewage discharge, and alteration of sedimentation regimes may be the main drivers of change, over and beyond the general pressure related to urbanized (URB) and cultivated areas (AGR).

This is not surprising since artisanal farming mostly represents agriculture in the study area. Urbanization is widely recognized as one of the strongest drivers impairing environmental conditions of coastal waters through alterations of the coastline, increased nutrient load, and/or inputs of pollutants [36,37]. However, in our case urbanization consist of small urban settlements mostly populated in summer, with a likely limited potential to cause relevant environmental modifications generally ascribed to this threat in other socio-ecological contexts [16,38].

Human activities on sandy shores (beach nourishments, deployment of artificial structures, etc.) may disrupt natural hydrodynamic and sedimentary regimes, which, in turn, could have detrimental effects on neighbouring rocky assemblages as a consequence of increased sandy bottom instability, alterations of sedimentation rates, and increased water turbidity [23,25]. Similar effects might be ascribable to the construction of coastal infrastructure, such as marinas, harbours, sewage outfalls [38] that, in addition, often represent also important sources of eutrophication and pollution [20,39–41]. Marine assemblages exposed to these pressures generally respond with a reduction in their complexity and an increased homogenization due to a decreased diversity, and the dominance of few opportunistic taxa such as turf-forming, filamentous or ephemeral seaweeds, or mussel beds [20,23,32,39,42].

Sessile assemblages subject to a general decrease of pressure levels (T3 and T5) showed complex three-dimensional structure, high taxon richness and β-diversity in both investigated habitats. Well developed *Cystoseira* canopies (always higher than 50% coverage, Fig 3C) and invertebrate taxa of the associated understorey were present in the lower midlittoral, whereas the shallow infralittoral was characterized by a suite of invertebrates and photophilic algae typical of shallow Mediterranean rocky substrates [20,43]. In this habitat, however, the remarkable presence of encrusting invertebrates and algae seems to indicate that the recovery from past disturbance related to the date-mussel collection, which led to the desertification of large extensions of rocky substrates in the infralittoral [18], is still incomplete. Clear changes to the structure observed for T3 and T5 were noticed in assemblages subject to higher levels of pressure (T1 and T4), which exhibited a decrease in α and β-diversity in both habitats. Turf-forming species were dominant at T1, whereas the presence of indicators of eutrophic conditions (such as opportunistic algae like *Gelidium* spp., *Colpomenia sinuosa* [20,44]) characterized assemblages at T4.

Such findings reinforce the assumption that co-occurring threats increase the vulnerability of marine ecosystems, which underlies current attempts for large-scale assessments of human pressure on oceans and seas [1,9]. These approaches, however, assign a cumulative impact score to a given spatial unit by weighting each pressure based on putative effects decided by expert judgement, which, indeed, might lead to under- or overestimate actual impacts. For instance, the medium-to-high impact scores that characterized the whole investigated area following Micheli et al. [9] were not confirmed, since we found large extensions of dense *Cystoseira* canopies in many sectors of coast. Also, the occurrence of a single strong pressure, which likely leads a given spatial unit to be classified under low impact, as coast sectors from T4 (see [1]), can nevertheless determine significant changes in assemblages. More importantly, the spatial resolution used to quantify the distribution of threats, and the ensuing response of marine communities, dictates the dimension of management strategies. Efforts to map cumulative impact at basin scale may provide a representation of large-scale patterns of vulnerability or help in setting priority areas for conservation and monitoring, thus informing management only from a general perspective. Such broad management frameworks should be then downscaled into context-specific actions and guidelines, which need local-scale refinements in the assessment of potential impacts and underlying pressures. The achievement of conservation targets through the implementation of MPAs provides an emblematic example to understand the complementarity between the two approaches. Large-scale assessments of human threats can identify potential sites for conservation highlighting areas with limited pressure, reduced socio-ecological conflicts, and pristine, or semi-pristine, environmental conditions, but local scale evaluations are important when MPAs are embedded in human-dominated seascapes. Our results show that, despite the presence of a MPA, human disturbance affects to some extent all areas in this stretch of coast. This situation commonly occurs [17] whenever MPAs cannot hold back pressures that originate outside their boundaries such as pollution, sediment load, climate change [45]. In these cases, the analytical approach adopted here, through high-resolution quantifications of threats and associated responses of marine communities, represents a step forward for the identification of replicated coastal sections featured by similar combination of threats and ensuing effects on marine systems, which are strictly necessary for spatial re-arrangement of protection regimes within MPAs, the identification of potential sites for restoring lost habitats, or to set actions aiming at reducing main pressures, in order to maximize conservation efforts.

## Conclusions

A complex scenario emerged from this analysis of interactions between anthropogenic activities and marine communities, which reflects conflicts and paradoxes typical of the whole Mediterranean Sea [8]. Our results demonstrate that the assessment of habitat vulnerability based on an expert opinion can be a practical solution in large-scale evaluations of potential effect of human pressure, but is probably unrepresentative of the actual vulnerability at local scale [11]. Understanding the relationships between human activities and their ecological impacts and assessing the spatial distribution of these impacts are crucial steps in the managing and conservation of marine species and habitats to maximize the use of marine resources while minimizing habitat degradation. Costs and logistic constraints often impair the potential to combine quantitative information on the distribution and intensity of human activities with the response by ecological systems, necessary to test predictions about the effects of some human-driven impacts. Major efforts in this direction should integrate expert opinion with direct quantifications to find thresholds driving changes in marine coastal assemblages [9].

As the so-called "blue economy" is rising worldwide, being particularly supported by current European policies, it is presumable that in the next years marine ecosystems will experience an increasing development of sea-based human activities [46,47]. This "blue growth", at least at European scale, is projected as a sustainable process. The Good Environmental Status is one of the main targets of the Marine Strategy Framework Directive [48] and clearly defines the meaning of “sustainable” when evaluating the expansion of marine uses. In this perspective, the systematic assessment of the effects of natural and anthropogenic drivers of change on biological communities at local scale may represents a privileged approach to harmonize broad scale management strategies in order to achieve the effective sustainability of human exploitation of marine resources.

## Supporting Information

S1 Fig PCA and CLUSTER analysis on threats. (a) Principal Coordinate Analysis (PCA) on normalized threat variables. Axes explained the 73% of variation among sectors. Dotted line enclosed sectors characterized by comparable levels and combinations of threats based on CLUSTER analysis. Clusters include sectors with dissimilarity values ≤ 50%. Numbers indicates sectors as in Fig 1 (see main text). Threat combination 1 (T1) include Sector 2; Threat combination 2 (T2) include Sectors 0, 1, 3; Threat combination 3 (T3) include Sectors 4, 5; Threat combination 4 (T4) include Sector 6; Threat combination 5 (T5) include Sectors 7, 8. (b) CLUSTER analysis showing the two groups of sectors characterized by comparable proportions of sandy coast (SC). Sectors were clustered together to form groups if among-sector dissimilarity was ≤ 50%.
(DOCX)

S1 Table List of taxa. Summary of taxa recorded in each habitat.
(DOCX)

S2 Table List of threats. Summary of threat indicators considered in the analyses. The acronym of each indicator as reported in the text is also indicated.
(DOCX)
