# Supplementary material for: The Challenge of Planning Conservation Strategies in Threatened Seascapes: Understanding the Role of Fine Scale Assessments of Community Response to Cumulative Human Pressures
Source: PLoS One. 2016 Feb 12;11(2):e0149253. doi: 10.1371/journal.pone.0149253 (PMC4752299; doi:10.1371/journal.pone.0149253)
Supplement: S1 Fig — (a) Principal Coordinate Analysis (PCA) on normalized threat variables. Axes explained the 73% of variation among sectors. Dotted line enclosed sectors characterized by comparable levels and combinations of threats based on CLUSTER analysis. Clusters include sectors with dissimilarity values ≤ 50%. Numbers indicates sectors as in Fig 1 (see main text). Threat combination 1 (T1) include Sector 2; Threat combination 2 (T2) include Sectors 0, 1, 3; Threat combination 3 (T3) include Sectors 4, 5; Threat combination 4 (T4) include Sector 6; Threat combination 5 (T5) include Sectors 7, 8. (b) CLUSTER analysis showing the two groups of sectors characterized by comparable proportions of sandy coast (SC). Sectors were clustered together to form groups if among-sector dissimilarity was ≤ 50%. (DOCX) [file pone.0149253.s001.docx]

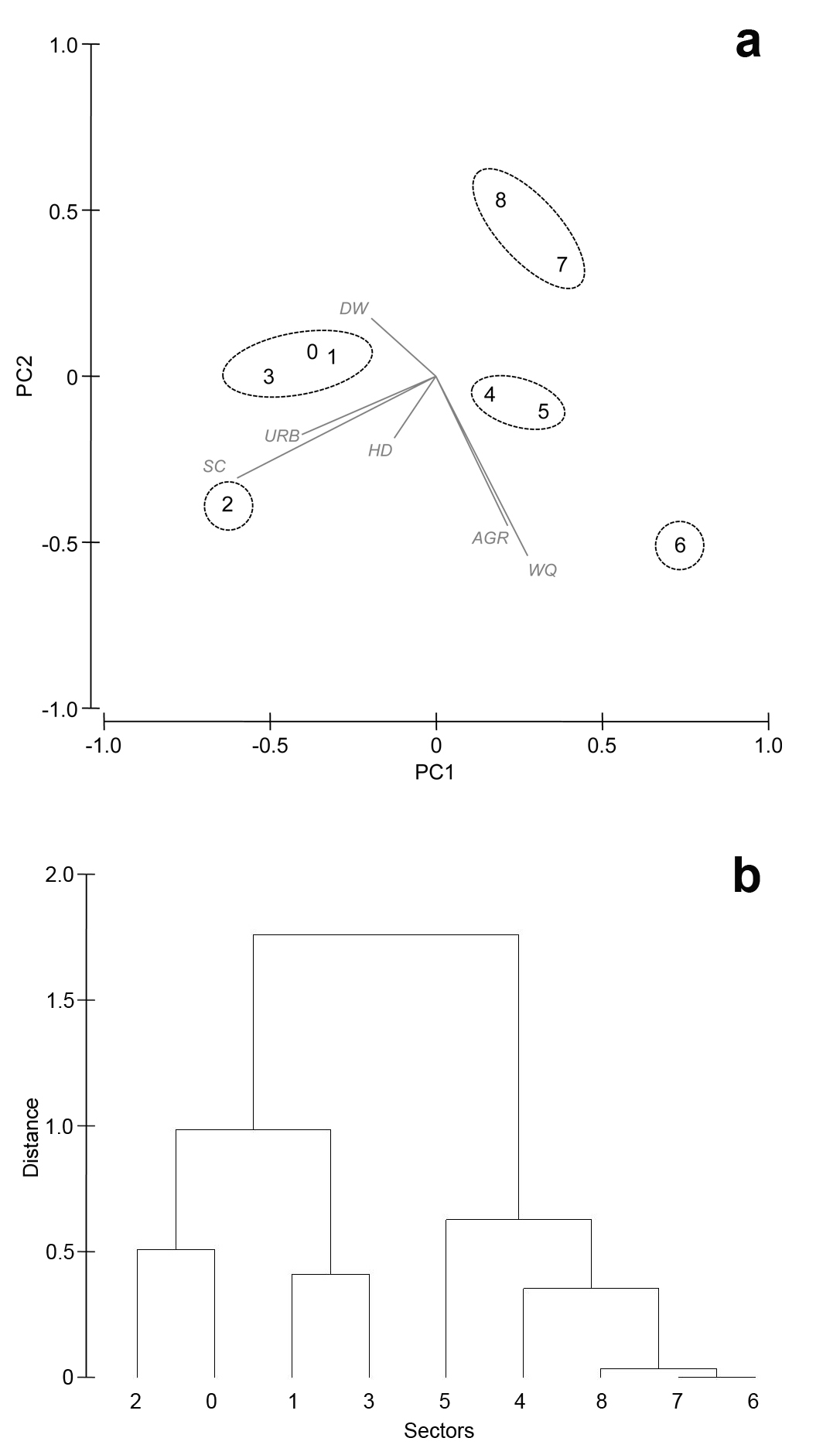


**S1 Fig. PCA and CLUSTER analysis on threats.** (a) Principal Coordinate Analysis (PCA) on normalized threat variables. Axes explained the 73% of variation among sectors. Dotted line enclosed sectors characterized by comparable levels and combinations of threats based on CLUSTER analysis. Clusters include sectors with dissimilarity values ≤ 50%. Numbers indicates sectors as in Fig 1 (see main text). Threat combination 1 (T1) include Sector 2; Threat combination 2 (T2) include Sectors 0, 1, 3; Threat combination 3 (T3) include Sectors 4, 5; Threat combination 4 (T4) include Sector 6; Threat combination 5 (T5) include Sectors 7, 8. (b) CLUSTER analysis showing the two groups of sectors characterized by comparable proportions of sandy coast (SC). Sectors were clustered together to form groups if among-sector dissimilarity was ≤ 50%.
