# Supplementary material for: The Challenge of Planning Conservation Strategies in Threatened Seascapes: Understanding the Role of Fine Scale Assessments of Community Response to Cumulative Human Pressures
Source: PLoS One. 2016 Feb 12;11(2):e0149253. doi: 10.1371/journal.pone.0149253 (PMC4752299; doi:10.1371/journal.pone.0149253)
Supplement: S1 Table — Summary of taxa recorded in each habitat. (DOCX) [file pone.0149253.s002.docx]

**S1 Table. List of taxa.** Summary of taxa recorded in each habitat.

| **Turf forming species** | **Habitat** | **Abbr.** | **Nitrophil algae** | **Habitat** | **Abbr.** | **Invertebrates** | **Habitat** | **Abbr.** |
| --- | --- | --- | --- | --- | --- | --- | --- | --- |
| *Acetabularia acetabulum* | IS | Ace | *Colpomenia sinuosa* | IS | Colp | Clavulariidae | I | Clav |
| *Amphiroa rigida* | IS | Amp | Gelidiales | IS | Gel | Clionidae | IS | Cli |
| *Anadyomene stellata* | IS | Ana | *Hypnea musciformis* | I | Hmu | *Corynactis viridis* | I | Cvir |
| Articulated Corallines | IS | AC | *Ulva* sp. | I | Ulv | *Cystodites dellechiajei* | S | Cdel |
| *Botryocladia* sp. | S | Botr | **Encrusting algae** |  |  | Didemnidae | IS | Did |
| *Caulerpa racemosa* | S | Cau | Bacteria | I | Bac | *Diplosoma listerianum* | IS | Dip |
| Coarsely branched algae | IS | CB | Cutleriales | IS | Cut | *Dysidea avara* | S | Dys |
| *Codium* sp. | S | Cod | Encrusting Calcifed Rhodophytes | IS | ECR | Encrusting bryozoans | IS | EB |
| *Corallina* sp. | IS | Cor | *Palmophyllum crassum* | IS | Pcr | Encrustiong red sponges | IS | ERS |
| Dark filamentous algae | IS | DFA | *Peyssonnelia* spp. | IS | Pey | *Gastrochaena dubia* | IS | Gdub |
| *Dasycladus clavaeformis* | S | Dcl | *Valonia macrophysia* | IS | Val | *Halocinthya papillosa* | S | Hpa |
| Dictyotales | IS | Dic | **Invertebrates** |  |  | *Hemimycale columella* | S | Hco |
| Dumontiaceae | S | Dum | *Actinia equina* | IS | Aeq | Hydroids | IS | Hyd |
| *Falkenbergia rufolanosa* | I | Fruf | Actinians | I | Act | *Ircinia* spp. | IS | Irc |
|  |  |  |  |  |  |  |  |  |
| **Turf forming species** | **Habitat** | **Abbr.** | **Invertebrates** | **Habitat** | **Abbr.** | **Invertebrates** | **Habitat** | **Abbr.** |
| *Flabellia petiolata* | S | Fpe | *Aiptasia mutabilis* | I | Amu | *Lithophaga lithophaga* | IS | Lit |
| *Gastroclonium clavatum* | I | Gcla | *Anemonia viridis* | IS | Avir | Massive dark sponges | IS | MDS |
| Green filamentous algae | IS | GFA | *Aplidium* sp. | S | Apl | *Microcosmus* sp. | S | Mic |
| *Halimeda tuna* | IS | Hal | *Aplysina aerophoba* | S | Aae | *Myriapora truncata* | S | Mtr |
| *Laurencia* complex | IS | Lau | *Arca noe* | I | Anoe | *Mytilus mitilaster* | I | Myt |
| *Liagora viscida* | I | Lvi | *Ascidia mentula* | S | Ame | *Oscarella* sp. | S | Osc |
| *Padina pavonica* | IS | Pad | Barnacles | IS | Bal | *Pentapora* sp. | S | Pen |
| Soft Branched algae | I | SBA | *Balanophyllia europaea* | S | Beu | *Petrosia ficiformis* | S | Pfi |
| Stypocaulaceae | I | Sty | *Bothryllus* sp. | S | Both | *Phorbas* sp. | IS | Pho |
| Thin Tubular Sheet-like algae | IS | TTS | *Caryophyllia* sp. | S | Cary | *Phorbas tenacior* | S | Pte |
| *Triclocarpa fragilis* | S | Tfr | *Cereus pedunculatus* | IS | Cpe | Serpulids | IS | Ser |
| *Wrangelia penicillata* | IS | Wpe | *Chlathrina* sp. | IS | Chl | *Spondylus* | I | Spo |
| **Canopy forming algae** |  |  | *Chondrilla nucula* | S | Cnu | Terpios fugax | S | Tfu |
| *Cystoseira* spp. | IS | Cys | *Chondrosia reniformis* | S | Cre | Thin ramified bryozoans | S | TRB |
| *Sargassum* | I | Sar | *Cladocora caespitosa* | S | Clad | Vermetidae | IS | Ver |

I = Lower midlittoral, S = Shallow infralittoral. The acronyms adopted for CAP analyses are also reported (Abbr.).
