# Supplementary material for: The Challenge of Planning Conservation Strategies in Threatened Seascapes: Understanding the Role of Fine Scale Assessments of Community Response to Cumulative Human Pressures
Source: PLoS One. 2016 Feb 12;11(2):e0149253. doi: 10.1371/journal.pone.0149253 (PMC4752299; doi:10.1371/journal.pone.0149253)
Supplement: S2 Table — Summary of threat indicators considered in the analyses. The acronym of each indicator as reported in the text is also indicated. (DOCX) [file pone.0149253.s003.docx]

**S2 Table. List of Threats.** Summary of threat indicators considered in the analyses. The acronym of each indicator as reported in the text is also indicated.

| **Threat indicators** | **Description** | **Threat acronym** |
| --- | --- | --- |
| Proportion of urban surface within a 1 km radius inland for each sector | Surface dedicated to urban fabric in a sector divided by the corresponding total surface. | URB^a^ |
| Proportion of agriculture surface within a 1 km radius inland for each sector | Surface dedicated to cultivated areas in a sector divided by the corresponding total surface. | AGR^a^ |
| Proportion of sandy coastline | Length of sandy coastline divided by length of total coastline of the corresponding sector. | SC^a^ |
| Harbour incidence | Linear distance from the central sampling site in each sector and the main harbour of Porto Cesareo. | HD |
| Water quality | Colony Forming Unit (CFU) of *Escherichia coli* in 100 ml | WQ^b^ |
| Weighted index of rock damage due to date-mussel fishery | Direct assessment based on the size and frequency of disturbed patches in each sampled surface. | DW^c^ |

^a^Data source: Territorial Information System of Apulia Region (www.sitpuglia.it)

^b^Data source: Regional Agency for Environmental Prevention and Protection (http://www.arpa.puglia.it/web/guest/balneazione)

^c^Data source: Field survey (See section ‘Assessment of the distribution of threats’, for further details)
